# Rosy Beginnings: Studying Peroxisomes in *Drosophila*

**DOI:** 10.3389/fcell.2020.00835

**Published:** 2020-08-25

**Authors:** C. Pridie, Kazuki Ueda, Andrew J. Simmonds

**Affiliations:** Department of Cell Biology, Faculty of Medicine and Dentistry, University of Alberta, Edmonton, AB, Canada

**Keywords:** *Drosophila melanogaster*, peroxisomes, embryo development, lipid metabolism, reactive oxygen species

## Abstract

Research using the fruit fly *Drosophila melanogaster* has traditionally focused on understanding how mutations affecting gene regulation or function affect processes linked to animal development. Accordingly, flies have become an essential foundation of modern medical research through repeated contributions to our fundamental understanding of how their homologs of human genes function. Peroxisomes are organelles that metabolize lipids and reactive oxygen species like peroxides. However, despite clear linkage of mutations in human genes affecting peroxisomes to developmental defects, for many years fly models were conspicuously absent from the study of peroxisomes. Now, the few early studies linking the Rosy eye color phenotype to peroxisomes in flies have been joined by a growing body of research establishing novel roles for peroxisomes during the development or function of specific tissues or cell types. Similarly, unique properties of cultured fly Schneider 2 cells have advanced our understanding of how peroxisomes move on the cytoskeleton. Here, we profile how those past and more recent *Drosophila* studies started to link specific effects of peroxisome dysfunction to organ development and highlight the utility of flies as a model for human peroxisomal diseases. We also identify key differences in the function and proliferation of fly peroxisomes compared to yeast or mammals. Finally, we discuss the future of the fly model system for peroxisome research including new techniques that should support identification of additional tissue specific regulation of and roles for peroxisomes.

## Overview

Peroxisomes are organelles found in almost all eukaryotic cells. Some functions of peroxisomes, especially in terms of oxygen and lipid metabolism can overlap those of mitochondria. However, the structure of peroxisomes and mitochondria are quite different with peroxisomes being composed of a single bilayer membrane surrounding a dense matrix composed of enzymes. The volume or number of peroxisomes within a single cell changes dynamically depending on surrounding conditions. There are several competing models for the mechanisms mediating peroxisome proliferation in mammalian cells, but all propose templated assembly either by fission and growth of the existing pool and/or *de novo* assembly from small membrane vesicles and proteins imported from the cytoplasm (reviewed in Mast et al., 2020).

Peroxisome composition and activity varies between organisms and even between cell types in multicellular animals. Generally peroxisomes are responsible for catabolism of branched or VLCFAs, purine catabolism, synthesis of specialized fatty acids like plasmalogens, ether-lipids and bile acids, and regulation of hydrogen peroxide and other ROS (reviewed in Fujiki et al., 2020). Yeast can survive without peroxisomes when grown in most conditions, but are dependent on their activity for β-oxidation (reviewed in Yuan et al., 2016). In mammals, β-oxidation occurs largely in the mitochondria. Peroxisomes are normally required for catabolism of branched and very-long chain fatty acids (≥C22) but can metabolize other fatty acids when mitochondria are compromised (Violante et al., 2019). Peroxisomes are functionally, and in some cases can be physically, linked to other organelles including the endoplasmic reticulum (ER), mitochondria, lipid droplets and lysosomes (reviewed in Schrader et al., 2020). Mutation in human genes encoding peroxisome assembly factors (Peroxins), or in enzymes within peroxisomes, leads to a variety of disorders ranging from mild to very severe forms causing death in infants (reviewed in Schiller et al., 2020).

Use of the fruit fly *Drosophila melanogaster* as a laboratory animal originated at the turn of the 20th century (reviewed in Tolwinski, 2017). Similar to mammals, *Drosophila* has a diploid genome with X (female) and Y (male) sex chromosomes. However, relative to mammals, the fly genome is less complex with approximately 15,500 genes on four chromosomes (Morgan and Bridges, 1916; Adams et al., 2000; Roy et al., 2010). The continued success of *Drosophila* for fundamental genetic and cell physiological research has been supported by the ongoing development of unique tools and collaborative efforts that have generated thousands of unique strains and reagents for genetics, cell and molecular biology studies (reviewed in St Johnston, 2002; Hales et al., 2015). This genetic tractability and a large innovative community of fly researchers remain key elements to its enduring laboratory use. Forward genetic screens driven by traditional mutagenesis techniques like X-rays, chemical mutagens and transposable element insertion or now CRISPR-Cas9, represent a well-used approach to identifying genetic pathways related to cell or developmental functions in flies (reviewed in Gratz et al., 2015). These screens are often part of multi-center, large-scale collaborative projects that generate a wealth of strains with unique mutations, most of which are made widely available from central stock centers (Bellen et al., 2004; Venken et al., 2011). One technique of particular note, used for many of the studies of fly peroxisomes, has been the generation of a large library of transgenic flies with selective time/tissue specific expression of the *S. cerevisiae Gal4* gene. These well characterized lines are used to drive transgenes containing an UAS. This binary expression regulation system permits facile alteration of gene expression by double-stranded (dsRNA) knockdown, cell ablation, ectopic gene expression, or expression of reporter genes within specific cell lineages within developing organs, especially the CNS (Brand and Perrimon, 1993; Pfeiffer et al., 2008).

Finally, although *Drosophila* is traditionally thought of as a laboratory animal used for genetic studies, there are also cultured cell lines available to complement whole animal studies. Most *Drosophila* cell lines are isolated by protease digestion or mechanical separation of tissues and immortalized spontaneously from primary cultures (Schneider, 1972; Ui et al., 1987). The most commonly used are Schneider 2 (S2) cells. These are transformable with standard methods, and are used to produce recombinant proteins and for *in vivo* imaging. S2 cells are particularly amenable to transient dsRNA-based RNA interference (RNAi; reviewed in Baum and Cherbas, 2008) and have been used extensively to identify genes involved in fundamental cell biological events including intracellular peroxisome movement (Kural et al., 2005).

## *Drosophila* Development

Early fly embryo development is largely dependent on mRNA and proteins supplied maternally, so the early stages of development can often proceed for some time even if the embryos are homozygous for mutations in essential genes (reviewed in Lasko, 2020). Under optimal conditions, embryogenesis completes 20–24 h after egg laying and a larva emerges. *Drosophila* larvae pass through three juvenile developmental stages, or “instars.” The first and second instars last 24 h each and the third instar lasts 48 h (Demerec, 1950). At the end of the third instar, the larva forms a pupa. During this stage much of the larval body is broken down and much of the adult (imago) develops *de novo* (Hales et al., 2015). Metamorphosis into the imago usually takes 84 h (Demerec, 1950; Hartenstein, 1993). Many adult tissues are pre-figured in the larval stage as collections of cells called imaginal disks. During metamorphosis, the imaginal disks form adult structures including antennae, limbs, eyes, genitals, and wings (reviewed in Beira and Paro, 2016). Most embryonic and larval organs are lost during metamorphosis but some, notably the CNS, are instead remodeled and are largely preserved into adulthood (Hartenstein, 1993). Adult flies have a lifespan of 45–60 days depending on genetic background and culture conditions (Demerec, 1950).

## *Drosophila* as a Model for Human Genetic Diseases

*Drosophila* has a simple body plan and organs analogous to most of those affected in human patients with peroxisomal disorders making them well-suited to studying the developmental effects of gene mutations that alter peroxisome function. Embryonic studies in particular are facilitated by the external development of the fly embryo, making it amenable to advanced imaging analyses (reviewed in Pantazis and Supatto, 2014). Many of the pathways that regulate development of analogous tissues in humans were characterized initially in *Drosophila* (reviewed in Wangler et al., 2015). Conservation of the pathways regulating early organ development between *Drosophila* and humans is sufficient in the CNS and PNS, musculature, kidney and liver analogs and lipid storage (fat body) for easy comparison of effects (reviewed in Yamamoto et al., 2014).

### Studying CNS/PNS Development and Behavior in Flies

Peroxisome diseases have a particularly strong effect on CNS development (reviewed in Faust et al., 2005). *Drosophila* has historically been valuable to model human diseases affecting the CNS. Some good examples include models of Alzheimer’s, Parkinson’s, Fragile X-syndrome, autism spectrum disorder and ALS (Phillips et al., 1995; Torroja et al., 1999; Feany and Bender, 2000; Wan et al., 2000; Whitworth et al., 2005; Park et al., 2006; Feiguin et al., 2009; Stessman et al., 2016; Ordonez et al., 2018). Flies have simple brain morphology and can exhibit learning and memory behaviors. As such, they can be used to study cognitive disorders (reviewed in Androschuk et al., 2015). The relative simplicity of fly behaviors, for example coordinated movement, larval food foraging and adult climbing or “negative geotaxis” or “bang assay” permit quantification because defects are easy to see (Le Bourg and Lints, 1992; Günther et al., 2016). Morphological and molecular characterization of CNS/PNS patterning, locomotor activity in embryos and larvae as well as behavioral assays like the climbing assay have been used to examine the effects of aberrant peroxisome function on CNS/PNS development in *Drosophila* (Faust et al., 2014; Bulow et al., 2018; Di Cara et al., 2018, 2019).

### The Fly Analogs of Kidney and Liver and Their Role in Lipid Metabolism

Peroxisome disorders in humans are associated with several deleterious effects on the organs responsible for lipid metabolism such as the kidney or liver (reviewed in Wanders, 2013). *Drosophila* can be used to model the effect of gene mutations on development and function of the human kidney and liver (reviewed in Bharucha, 2009). The *Drosophila* Malpighian tubules are functionally analogous to the renal tubules of the vertebrate kidney, clearing toxins, and regulating ion homeostasis in the hemolymph (reviewed in Gautam et al., 2017). Nephrocyte cells, surrounding the heart (pericardial) and esophagus (garland), combined with the Malpighian tubule perform the size-based filtration mechanism of podocyte cells found in the glomerulus of the mammalian kidney (reviewed in Weavers et al., 2009). One good example of modeling kidney disease in flies is xanthinuria type one caused by loss of enzyme xanthine dehydrogenase (XDH). This results in yellowish inclusions indicating xanthine stone, xanthine and hypoxanthine accumulation, and low levels of metabolites like uric acid (Dent and Philpot, 1954; Hadorn and Schwinck, 1956; Hilliker et al., 1992). In humans, XDH has been proposed to localize to peroxisomes and the cytosol, but in *Drosophila* it is exclusively peroxisomal (Angermüller et al., 1987; Beard and Holtzman, 1987; Ichikawa et al., 1992). Flies have also been used previously to model the effects of dysfunction of a single peroxisome enzyme. Urate oxidase (Uro), an enzyme catalyzing the generation of uric acid following XDH activity, is present in peroxisomes in *Drosophila* and mammals. However, Uro is inactive in primates (Wu et al., 1989). Knockdown of the *Uro* gene in Malpighian tubules recapitulates uric acid nephrolithiasis, reduced longevity, and resulted in purine sensitivity (Lang et al., 2019).

Modeling the effects of specific gene mutations on liver function is somewhat more complicated in flies. While *Drosophila* fat body was long considered functionally equivalent to the liver, oenocytes are now considered by most to be the functional equivalent of hepatocytes based on similarities in lipid metabolism (reviewed in Makki et al., 2014). In insect models of mammalian liver function the contribution of both the fat body and oenocytes must be taken into account. Oenocyte-specific mutations and knockdown, as well as dietary approaches, have been used to successfully model non-alcoholic fatty liver disease (reviewed in Ugur et al., 2016). The lipid mobilization ability of the *Drosophila* fat body has been used to model human metabolic diseases associated with abnormal lipid storage, such as obesity linked to the lipases and perilipins caused by loss of *brummer* (*bmm*) and *Lipid storage droplet-1* (*Lsd-1*), or *Lipid storage droplet-2* (*Lsd-2*) overexpression, respectively; liver steatosis, linked to mutation in *bmm* overexpression in fat body and non-alcoholic fatty liver disease, in *Drosophila Hepatic nuclear factor 4* (*Hnf4*)-null flies via genetic or dietary methods (Grönke et al., 2003; Gutierrez et al., 2007; Beller et al., 2010; Ugur et al., 2016). Mutations in peroxisome-linked genes have significant effects on the development or function of Malpighian tubules, fat body and oenocytes (Beard and Holtzman, 1987; Faust et al., 2014; Bulow et al., 2018; Huang et al., 2019).

## Rosy Eyes and Other Early Studies of Mutations Affecting *Drosophila* Peroxisomes

**FIGURE 1:**
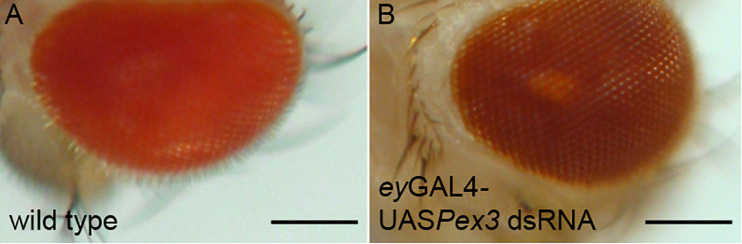
Knockdown of *Pex3* in the *Drosophila* eye phenocopies the Rosy phenotype. **(A)** Wild type *Drosophila* eye pigment is a bright red-orange color. **(B)** Reduction of Pex3 activity specifically in the eye by a combination of *eyeless*(ey) GAL4 and a UAS inducible transgene expressing a dsRNA targeting *Pex3* mRNA causes the characteristic dark red pigmentation associated with *ry* mutations. Bar = 300 μm.

The earliest examples of peroxisome studies in *Drosophila* were electron micrographs observing the localization of the peroxisomal enzymes XDH, encoded by *rosy* (*ry*), and D-amino acid oxidase (DAO; Beard and Holtzman, 1987; St Jules et al., 1989, 1990). Beard and Holtzman (1987) were the first to show that a *Drosophila* peroxisomal enzyme had tissue-specific differences in expression. Using 3,3′-diaminobenzidine-based detection of catalase suitable for TEM imaging they determined that peroxisomes were abundant in wild-type *Drosophila* Malpighian tubules and gut. EM-based visual examination of the peroxisomes in adult *Drosophila* heads determined peroxisome abundance varied with tissue type, suggesting the factors that govern their biogenesis and function might be tissue specific (St Jules et al., 1990). Flies homozygous for *ry* loss-of-function alleles lacked detectable XDH activity and had reduced catalase activity (Beard and Holtzman, 1987). *Drosophila* XDH activity requires functional peroxisomes as the Rosy eye phenotype also occurs when *Peroxin* (*Pex*) gene function is inhibited in the developing eye (Figure 1) (Nakayama et al., 2011). Notably, many of these early papers directly commented on the suitability of *Drosophila* for the study of heritable peroxisome disorders, and proposed a link between peroxisomes and other organelles based on observations of increased peroxisomal abundance in cells of the fat body (St Jules et al., 1990). Despite this endorsement, flies were not widely utilized as a model system for studying peroxisomes for another 20 years.

## Identifying and Characterizing *Drosophila Pex* Proteins

**FIGURE 2:**
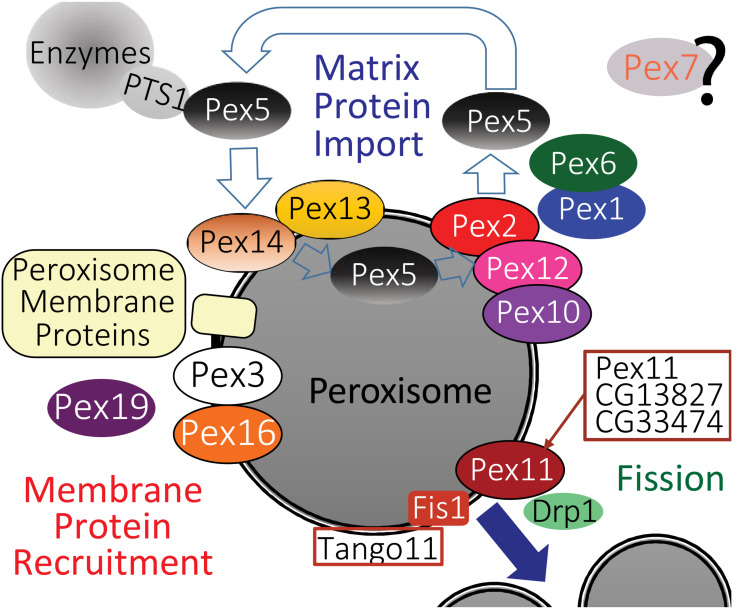
A model for the likely role of the *Drosophila* homologs of peroxisome biogenesis factors, based on a recent one for mammalian peroxisome proteins by Fujiki et al. (2020). Homologs of Pex proteins (Table 1) required for recruitment and insertion of peroxisome membrane proteins (Table 2), as well as Pex3, Pex16, and Pex19 are all conserved in *Drosophila*. The peroxisome fission machinery also appears conserved as there are three *Drosophila* Pex11 proteins (Pex11, CG13827, and CG3374). *Fis1* is the homolog of *Fission, mitochondrial 1* (*FIS1*), *Tango11* is homologous to *Mitochondrial fission factor* (*MFF*) and *Drp1* is the homolog of *Dynamin 1 like* (*DNM1L*), all of which are required for peroxisome fission in mammals. *Drosophila* peroxisomal matrix protein import in flies appears dependent on PTS1, mediated by Pex5 binding to PTS1 (Table 3), although some matrix proteins do not have a PTS1 motif (Table 4). The docking complex proteins Pex13 and Pex14 are conserved in *Drosophila*, as is the RING-E3-ligase complex of Pex2, Pex10, and Pex12. It is therefore likely that Pex5-bound cargos are imported, then Pex5 is recycled to the cytosol by Pex1 and Pex6 in a manner similar to mammals. A Pex7 homolog exists in *Drosophila*, but its role in peroxisome function is unclear.

Pex proteins play key roles in peroxisome proliferation (Figure 2) including: forming the vesicles that supply new peroxisome membrane; inserting proteins into the peroxisome membrane including the transmembrane pores through which enzymes are recruited into the peroxisome matrix; recycling of Pex proteins that direct enzymes into peroxisomes and fission of existing peroxisomes (reviewed in Mast et al., 2020). The source of vesicles that supply membrane to new/growing peroxisomes in yeast cells is the ER (Hoepfner et al., 2005). In mammalian cells the source of vesicles supplying membrane to proliferating peroxisomes is controversial, with ER and/or mitochondria origin proposed (Rucktaschel et al., 2010; Kim, 2017; Sugiura et al., 2017). In mammalian cells, Pex13 and Pex14 are inserted post-translationally into the membrane by the combined action of Pex3 and Pex19 to form the “importomer” complex, which, combined with the activity of Pex2, Pex10, and Pex12 provides the pore through which enzymes are imported into the matrix. Cargos are recognized and carried through the importomer by Pex5, which is then recycled back to the cytoplasm by the activity of Pex1/Pex6 (reviewed in Fujiki et al., 2020). Most enzymes that are recognized by Pex5 and directed to the importomer have a variant of the PTS1 (serine–lysine–leucine, SKL) at their C-terminal end. Yeast and mammalian cells have a second peroxisome-targeting signal, PTS2 is present on some peroxisomal enzymes (e.g., thiolase) which is recognized by PEX7 which binds a variant of PEX5 (Glover et al., 1994; Subramani, 1998).

**TABLE 1:** *Drosophila* homologs of human Pex proteins.

**Human**	**Role**	***Drosophila***	**Loc**	**Func**	**Protein interactions**
PEX1	Protein import	Pex1	(+)^B^	M	
PEX2	Protein import	Pex2	(+)^B^	M	
PEX3	Membrane assembly	Pex3	(−)^B^	M	Pex19 (FlyBi)
PEX5	Protein import	Pex5	(+)^B^	M	Pex14 (G)
PEX6	Protein import	Pex6	(+)^B^	M	
PEX7	Protein import	Pex7	(+)^B^		
PEX10	Protein import	Pex10	(+)^B^		
PEX11A/B	Fission	Pex11	(+)^B^	M	
PEX11C	Fission	CG13827	(+)^B^		
PEX11C	Fission	CG33474	(+)^B^		
PEX12	Protein import	Pex12	(+)^B^	M	
PEX13	Protein import	Pex13	(+)^B^	M	
PEX14	Protein import	Pex14	(+)^B^	M	Pex5 (G)
PEX16	Membrane assembly	Pex16	(+)^B^	M	
PEX19	Membrane assembly	Pex19	(+)^B^	M	Pex3 (FlyBi)

*“Loc”: (+) validated localization of the protein to the peroxisome, (−) did not localize to peroxisomes in B-(Baron et al., 2016). “Func”: experimental confirmation of an effect on peroxisome proliferation in fly cells M-(Mast et al., 2011). Protein interactions indicate interaction between these protein partners in large-scale yeast 2-hybrid (FlyBi) or (G) whole proteome mass-spectrometric identification (Guruharsha et al., 2011).*

**TABLE 2:** *Drosophila* homologs of known peroxisomal membrane proteins.

**Human**	**Role**	***Drosophila***		**Loc**
ABCD1/2	Fatty acid transporter	ABCD	ATP binding cassette subfamily D	(+)^B^
ABCD3	Fatty acid transporter	Pmp70	Peroxisomal membrane protein 70 kDa	(+)^B^
ATP2C1	Ca^2+^ transporter	SPoCk	Secretory pathway calcium atpase	(−)^B^
BEST4	Channel	Best2	Bestrophin 2	(+)^*C*^
FAR1/2	Ether lipid synthesis	CG5065	Fatty acyl-CoA reductase	(+)^B^
FIS1	Peroxisome fission	Fis1	Fission, mitochondrial 1	(−)^B^
MP17L	ROS metabolism	CG12355		(−)^B^
MVP17/PMP22/PXMP2	Channel	CG11077		(+)^B^
SLC27A1/4	Fatty acid transporter	Fatp1	Fatty acid transport protein 1	(−)^B^
SLC22A5	Carnitine transporter	Orct	Organic cation transporter	(+)^B^
SLC25A17	Transporter	PMP34	Peroxisomal membrane protein 34	(+)^B,F^
TMEM135		CG11737		(+)^B^
MFF	Fission	Tango11	Transport and Golgi organization 11	

*“Loc”: (+) experimentally validated localization to peroxisomes, (−) did not localize to peroxisomes in B-(Baron et al., 2016), F-(Faust et al., 2012) or (C-Chintapalli et al., 2012).*

**TABLE 3:** *Drosophila* homologs of human enzymes with PTS1 motifs.

**Human**	***Drosophila***	**Function**	**Enzyme**	**Interactions**	**Loc**
ACOX1	CG5009	β-Oxidation	Acyl-CoA oxidase	Cat(FlyBi)	(+)^B^
ACOX3	CG9527	β-Oxidation	Acyl-CoA oxidase		(+)^B^
ACSF2	CG12512	β-Oxidation	Long-chain-fatty-acid-CoA ligase		(+)^B^
AGPS	ADPS	Ether lipid synthesis	Alkyldihydroxyacetone-phosphate synthase		(+)^*B,F*^
AGXT	Spat	Amino acid metabolism	Serine pyruvate aminotransferase		(+)^B^
AMACR	Amacr	α-Oxidation	Alpha-methylacyl-CoA racemase		(+)^B^
CAT	Cat	ROS metabolism	Catalase	CG5009(FlyBi)	(+)^B^
CCS	Ccs	ROS metabolism	Copper chaperone for superoxide dismutase	Giot1 (G)	(+)^B,F^
CRAT	CRAT	β-Oxidation	Carnitine *O*-acetyltransferase		(+)^B^
CROT	CROT	β-Oxidation	Carnitine *O*-octanoyltransferase		(+)^B^
DAO	CG11236	Amino acid metabolism	D-Amino-acid oxidase		(+)^B^
DDO	CG12338	Amino acid metabolism	D-Amino-acid oxidase		(+)^B^
DHSR4/PECR	CG10672	β-Oxidation/α-oxidation	Carbonyl reductase (NADPH) 3-beta-hydroxysteroid		(+)^B^
			3-dehydrogenase		
ECH1	CG9577	β-Oxidation	Delta(3,5)-Delta(2,4)-dienoyl-CoA isomerase		(+)^B^
ECI2	Dci	β-Oxidation	Dodecenoyl-CoA delta-isomerase		(+)^B^
GNPAT	Dhap-at	Ether lipid synthesis	Dihydroxyacetone phosphate acyltransferase		(+)^B^
GOT1	Got1	Amino acid metabolism	Glutamate oxaloacetate-transaminase	Ccs, Sod1 (G)	(+)^B^
HACL1	Hacl	α-Oxidation	2-Hydroxyacyl-CoA lyase		(−)^B^
HADHA/LBP	Mtpα	β-Oxidation	Mitochondrial trifunctional protein α subunit		(+)^B,F^
HAO1/2	CG18003	Hydroxyacid oxidase	L-Lactate dehydrogenase (S)-2-hydroxy-acid oxidase		(+)^B^
HSD17B4/DBP	Mfe2	β-Oxidation	Multifunctional enzyme type 2	Mfe2(H)	(+)^B^
HSDL2	CG5590	Hydroxysteroid dehydrogenase	Hydroxysteroid dehydrogenase		(+)^B^
IDE	Ide	Protease	Insulin degrading metalloproteinase		(−)^B^
MDH1	Mdh1	α-Oxidation	Malate dehydrogenase		(+)^B^
SCP2	CG17597	β-Oxidation	Thiolase		(+)^B^
SOD1	Sod1	ROS metabolism	Superoxide dismutase	Got1(G)	(+)^B,F^
TYSND1	CG3589	Protease (Removal of PTS1 signal)	Peroxisomal leader peptide-processing protease		(+)^B^
UOX*	Uro	Purine metabolism	Urate oxidase		(+)^B^

“Interactions”: Protein interactions indicate interaction between these protein partners in G) whole proteome mass-spectrometric identification G-(Guruharsha et al., 2011) or experimentally by H-(Haataja et al., 2011). “Loc”: (+) experimentally validated localization to peroxisomes, (−) did not localize to peroxisomes in B-(Baron et al., 2016) F-(Faust et al., 2012). *UOX is a pseudogene in primates.

**TABLE 4:** *Drosophila* peroxisome matrix enzymes without a PTS1 motif.

**Human**	***Drosophila***	**Function**	**Function**	**Loc**
ACAA1	CG9149	β-Oxidation	β-Ketoacyl-CoA thiolase	(−)^B^
ACAD11	CG4860	β-Oxidation	Short-chain acyl-CoA dehydrogenase	(−)^B^
ACOX2	CG17544	β-Oxidation	Acyl-CoA oxidase	(+)^B^
ACSL1	CG3961	β-Oxidation	Long-chain-fatty-acid–CoA ligase	(−)^B^
ACSL3/4	Acsl	β-Oxidation	Acyl-CoA synthetase long-chain	(−)^B^ (+)^H^
DECR2	CG3699	β-Oxidation	17-Beta-estradiol 17-dehydrogenase	(−)^B^
DRS7B	CG31548	β-Oxidation	17-Beta-estradiol 17-dehydrogenase	(−)^B^
EPHX2	Pummelig	ROS metabolism	Carboxylesterase	(−)^B^ (+)^HT^
HMGCL	Hmgcl	Amino acid metabolism	Hydroxymethylglutaryl-CoA lyase	(+)^B^
HMGCR	Hmgcr	Isoprenoid synthesis	HMG coenzyme A reductase	(+)^B^
IDI1/2	Idi	Isoprenoid synthesis	Isopentenyl-diphosphate delta-isomerase	(−)^B^
IDH1	Idh	α-Oxidation	Isocitrate dehydrogenase	(−)^B^
LONP	Lon	Protease	Lon protease	(+)^B^
MARF1	Marf1	mRNA stability	Meiosis regulator and mRNA stability	(−)^B^
NUDT7	CG11095		Coenzyme A diphosphatase	(−)^B^
PAOX	CG8032		Spermine/spermidine oxidase	(−)^B^
PHYH	CG14688	α-Oxidation	Phytanoyl-CoA dioxygenase	(−)^B^
PMVK	CG10268	Isoprenoid synthesis	Phosphomevalonate kinase	(+)^B^
PRDX1	Jafrac	ROS metabolism	Peroxiredoxin	(−)^B^
PRDX5/PMP20	Prx5	ROS metabolism	Peroxiredoxin	(−)^B^
XDH	Ry	Purine metabolism	Xanthine dehydrogenase	(+)^B^
ACSF3	CG18155	β-Oxidation	Long-chain-fatty-acid–CoA ligase Malonate–CoA ligase	(+)^B^
NUDT19	CG10194		Coenzyme A diphosphatase	(+)^B^
MUL1	Mul1		Mitochondrial E3 ubiquitin protein ligase	(+)^B^
MVK	CG33671	Isoprenoid synthesis	Mevalonate kinase	(−)^B^
NOS2	Nos	ROS metabolism	Nitric oxide synthase	(+)^B^
SERHL/SEHL2	Kraken	Protease	Serine hydrolase-like	(−)^B^
SOD2	Sod2	ROS metabolism	Superoxide dismutase	(−)^B^

*“Loc”: (+) experimentally validated localization to peroxisomes, (−) did not localize to peroxisomes in B-(Baron et al., 2016), F-(Faust et al., 2012), H-(Huang et al., 2016) or HT-(Hehlert et al., 2019).*

The release of a largely complete *Drosophila* genome sequence in the early 2000s facilitated sequence-similarly based identification of genes and proteins homologous to those other organisms (Adams et al., 2000). This led to several publications identifying potential homologs of human or yeast PEX and other peroxisome-related proteins by predicted amino acid sequence homology (Chen et al., 2010; Mast et al., 2011; Faust et al., 2012). The fly genome was found to have several conserved *Pex* genes encoding proteins highly similar to their human or yeast counterparts. These included *Pex1*, *Pex2*, *Pex3*, *Pex5*, *Pex6*, *Pex7*, *Pex10*, *Pex11*, *Pex12*, *Pex13*, *Pex14*, *Pex16* and *Pex19* (Table 1) (Chen et al., 2010; Mast et al., 2011).

Confirmation of functional conservation of the import role of the predicted *Drosophila Pex* genes came first via RNAi-based knockdown screen in S2 cells stably expressing a GFP-peroxisomal targeting signal 1 (PTS1) fusion protein reporter that would be imported into peroxisomes with a functional importomer complex resulting in a punctate cytoplasmic GFP signal (Kural et al., 2005; Mast et al., 2011). The phenotypes induced by RNAi treatment targeting each putative *Pex* gene fell broadly into two categories. The first was cytosolic mislocalization of a GFP-PTS1 fusion protein (*Pex1*, *Pex2*, *Pex5*, *Pex6*, *Pex12*, *Pex13*, *Pex14*, *Pex16*, *Pex19*), identifying defects in protein import. The second category was aberrant peroxisome morphology/number (*Pex3*, *Pex11*), implying defects in proliferation. No effect on GFP-PTS1 transport into peroxisomes was observed for dsRNA knockdown of the gene homologous to *Pex7* (Mast et al., 2011). *Drosophila* genes with weak similarity to yeast *Pex20p* or *Pex23p* were also identified, but RNAi treatment targeting these two genes did not cause a defective peroxisome import phenotype (Mast et al., 2011).

## Identification of Other Peroxisome Factors

Identification of the *Drosophila Pex* homologs was followed by a homology based prediction of other peroxisome-linked genes such as those encoding peroxisomal membrane proteins, fission factors and matrix enzymes. Faust et al. (2012) identified multiple potential orthologs for many of the peroxisome proteins, based on the presence of a PTS1 domain or homology to known peroxisome proteins in yeast or mammals. Baron et al. (2016) systematically tested the peroxisome localization of each of these predicted peroxisomal proteins encoded by these, confirming the subset that was targeted to peroxisomes, and likely represented *bona-fide* peroxisome factors (Tables 2–4; reviewed in Anderson-Baron and Simmonds, 2018). Notably, in S2 cells, Pex3 and Pex19 were localized to peroxisomes at a level less than was expected, but functional tests suggested this was likely due to the epitope tags used for visualization (Baron et al., 2016). Functional conservation has also been demonstrated experimentally for Pex3. When expressed in S2 cells yeast *Pex3* was functional, and that the N-terminal domain of Pex3 directed sorting of other Pex proteins and peroxisome membrane proteins to ER sub-domains that likely represent a source of vesicles that are incorporated into peroxisomes (Fakieh et al., 2013).

Confirmation of conserved protein–protein interactions between fly Pex proteins has largely come from large-scale whole-proteome projects that were not looking at Pex proteins specifically (Tables 1, 3). One good example of these type of communal and open-science nature of the *Drosophila* community is the FlyBi project^1^, a large-scale effort to map all potential protein interactions in an unbiased yeast 2-hybrid screen with the goal of a whole proteome binary interaction network for *Drosophila*. Pex3 and Pex19 were shown to interact by FlyBi, suggesting they act to target peroxisome membrane proteins to the membrane in a manner similar to mammalian cells (Figure 2). Similarly, Pex5 and Pex14 were identified as interacting as part of a large scale mass spectrometry screen (Guruharsha et al., 2011), suggesting they conserve their respective functions as a cargo binding protein (Pex5) and import channel (Pex14) in *Drosophila* cells (Figure 2).

## *Drosophila* Has PEX7 but Not PTS2-Mediated Peroxisome Import

A major difference in peroxisome function between *Drosophila* and yeast or mammals is the absence of PTS2-mediated import. The majority of peroxisome matrix enzymes in *Drosophila*, including homologs of those with canonical PTS2-bearing cargo proteins in yeast or mammals (i.e., thiolase, SCPx), have a C-terminal PTS1 motif or conserved equivalent in flies (Table 3) (Faust et al., 2012). Analysis of the *Drosophila* proteome failed to detect the N-terminal PTS2 motif in any likely peroxisome trafficked proteins, and fluorescent microscopy data showed a PTS2-mCherry construct was unable to localize to peroxisomes in S2 cells (Faust et al., 2012). Supporting this, a reporter chimera of the putative PTS2-dependent alkylglycerone phosphate synthase homolog, encoded by *CG10253*, co-localized with the peroxisome marker PMP34-Cerulean only when the reporter was at the N-terminus, suggesting *CG10253* encoded a peroxisomal matrix protein with a C-terminal PTS (Faust et al., 2012).

Other species like *C. elegans* have also lost the PTS2 pathway but also lack a *Pex7* homolog (Motley et al., 2000). Although PTS2 mediated protein targeting cannot be identified in *Drosophila*, the presence of a *Pex7* homolog of raises questions as to its role in terms of peroxisomes. *Drosophila Pex7* is a functional homolog of mammalian PEX7 as it can substitute for human PEX7 to rescue PTS2-mediated peroxisome protein import in human *PEX7* mutant cells (Di Cara et al., 2019). A Pex7 reporter fusion partially localized to peroxisomes (Baron et al., 2016). *Pex7* was shown to be required for the ROS burst that precedes the *Drosophila* immune response (Di Cara et al., 2017). In terms of development, *Pex7* mutants show reduced viability and CNS defects. However, these defects are more mild than those linked to mutations in *Pex5* (Di Cara et al., 2019).

A clue for Pex7 activity in *Drosophila* may come from its pattern of expression. Compared to the relatively ubiquitous expression of *Drosophila Pex* genes in the early embryo *Pex7* is expressed at relatively high levels in only a small subset of cells thought to be of neural lineage (Pridie and Simmonds, 2020). This suggests a more specialized, potentially cell-lineage specific role. This role appears to be linked with peroxisome activity as phenotypes associated with targeted *Pex7* RNAi in the gut were rescued by ubiquitous *Catalase* over-expression. Also, loss of *Pex7* affects the overall redox state at the cellular and whole-organism levels (Di Cara et al., 2018). This suggests that *Pex7* plays a role in peroxisomal ROS management, affecting enzymes of the Catalase enzymatic pathway, e.g., SOD. The likely scenario is that *Drosophila Pex7* likely retains a peroxisome-related function unrelated to PTS2-mediated import. What this role could be remains unknown, but the unique situation in *Drosophila* where Pex7 is present but PTS2 import is not provides a unique platform to determine what it could be. It is particularly intriguing to consider that this putative Pex7 function outside of PTS2 cargo recognition may also be active in other organisms.

## Regulation of Peroxisome Volume or Number in *Drosophila*

Although localization of homologous *Drosophila* proteins to peroxisomes has been largely confirmed experimentally (Tables 1–4), conservation of their functional roles for the most part has not. This includes the mechanisms regulating peroxisome volume and number. In steady state conditions, homeostatic feedback mechanisms are thought to keep the relative volume and number of peroxisomes constant. However, peroxisome volume and number responds to changes to the cellular environment. This response can be increased volume or number, or reduction in peroxisome abundance via macroautophagy (reviewed in Chang et al., 1999; Germain and Kim, 2020). This mechanisms underlying pexophagy have been shown most clearly in yeast (Motley and Hettema, 2007). Peroxisome proliferation can be correlated to changes in expression of *Pex* genes as well as other peroxisome-linked genes. The volume and number of fly peroxisomes responds to changes in peroxisome-linked gene expression. S2 cells cultured under standard conditions have between 60 and 80 peroxisomes with an average volume of 173 nm^3^ (Baron et al., 2016). Altering the levels of *Drosophila* peroxisome factors, including Pex proteins, membrane proteins or peroxisomal enzymes often had significant effects on volume or number (Baron et al., 2016).

### Transcriptional Regulation of *Pex* and Peroxisome Related Genes

Regulation of the transcription of peroxisome-linked genes in *Drosophila* is currently not well characterized. Peroxisomes can be induced to proliferate by excess fatty acids and their analogs (reviewed in Yan et al., 2005). In mammalian cells, this occurs in part by inducing increased expression of genes encoding peroxisome proliferation factors regulated by conserved, lipid-sensing members of a nuclear hormone receptor superfamily called peroxisome-proliferator activated receptors (PPARs; reviewed in Bocos et al., 1995). Upon ligand binding, the three PPAR variants (α, β/δ, γ) form heterodimers with the retinoid X-receptor (RXR) to express enzymes involved in β-oxidation and other reactions to regulate lipid metabolism (reviewed in Kliewer et al., 2001). In mice, both PPARα and PPARγ regulate expression of *Pex* genes, however, the link between PPARs and peroxisome proliferation in humans is less clear (Dubois et al., 2017; Mirza et al., 2019).

*PPAR* homologs do not seem to be present in the *Drosophila* genome, but other nuclear hormone receptors likely act in a similar fashion. *Drosophila Hnf4* generally regulates β-oxidation in the larva (Palanker et al., 2009). *PPAR*α is highly expressed in liver and kidney and *Hnf4* is likewise highly expressed in the analogous fly tissues, the oenocytes and Malpighian tubules, respectively (Palanker et al., 2009). Both PPARα and Hnf4 are activated by LCFAs to induce expression of fatty acid β-oxidation enzymes (Göttlicher et al., 1992; Braissant et al., 1996; Palanker et al., 2009). The fly analog of PPARγ is less clear, but candidates may be inferred from phenotypes associated with their roles in the CNS. PPARγ is implicated in mitigating the neurodegenerative phenotype in ALS through its anti-inflammatory role (Li et al., 2000; Kiaei, 2008). Mice ALS models carrying mutations in *SOD1*, and *Drosophila* ALS models overexpressing TDP-43, both displayed ALS-like neurodegeneration that was rescued by the PPARγ agonist pioglitazone (Kiaei, 2008; Joardar et al., 2015). The phylogenetic relationship with human nuclear receptors predicts E75 and E78 of *Drosophila* as one of the closest potential PPARγ homologs (King-Jones and Thummel, 2005). Accordingly, in *Drosophila* ALS models, TDP-43 induction in glial and motor neurons, but not in musculature, was shown to rescue locomotor deficits upon agonist introduction when both E75 and E78 were present, suggesting functional conservation (Joardar et al., 2015). Notably, *spargel* (*srl*) the *Drosophila* ortholog of PPARγ co-activator 1α (PGC1-α) is upregulated in *Pex19* mutant cells (Bulow et al., 2018), suggesting that an analogous pathway plays a role in feedback regulation of peroxisome abundance.

### Effect of Peroxisome Enzyme Abundance on Peroxisomes

The effect of peroxisomal enzyme abundance on peroxisome volume or number is variable. Elevated levels of phosphomevalonate kinase (Pmvk), mitochondrial ubiquitin ligase activator of NF-κB (Mul1), carnitine *O*−octanoyltransferase (Crot) and α−methylacyl−CoA racemase (Amacr) lead to an average four-fold increase of normal peroxisome volumes. Conversely, Dhsr4 and ScpX lead to decreased peroxisome volume when overexpressed in S2 cells (Baron et al., 2016). The molecular mechanism underlying this decreased volume is likely indirect via feedback caused by altered metabolism of certain peroxisomal lipid species. One particular class of enzyme that responds to changes in lipid metabolism is fatty acyl-CoA reductase (FAR) family proteins. These participate in an early rate-limiting step of plasmalogen synthesis and the cellular levels of FAR proteins are directly dependent on plasmalogen level (Honsho et al., 2013). *FAR1* upregulation is observed in mice when plasmalogen synthesis was genetically inhibited, which was not associated with change in peroxisome abundance (Kimura et al., 2018; Merkling et al., 2019). *Drosophila* has 17 potential FAR orthologs of which only one has been examined experimentally. The one that has, *Sgroppino*, localizes to a subset of peroxisomes (Merkling et al., 2019). Accordingly, *Sgroppino* was not shown to affect *Drosophila* peroxisome number. However, mutations in genes encoding other peroxisome enzymes do have a strong effect on peroxisome proliferation in mammalian cells. For example, fibroblasts from patients with mutations in enzymes involved in β-oxidation pathway, acyl-CoA oxidase and multifunctional enzyme type 2/17-beta-hydroxysteroid dehydrogenase 4 (MFE-2/HSD17B4) show a discernible reduction in peroxisome abundance (Chang et al., 1999). Elevated expression of *Drosophila Mfe2* (a.k.a. *DBP*) did not seem to affect peroxisome volume but did reduce peroxisome abundance (Baron et al., 2016). Although, the ectopic overexpression of gain-of-function mutant form of human *ACOX1* in flies is reported to have no discernible effect on peroxisome numbers relative to overexpression of its wild type counterpart (Chung et al., 2020). Thus, the relationship between the relative abundance of various peroxisome enzymes and the volume or number of peroxisomes in *Drosophila* remains to be established.

### Regulation of Peroxisome Fission

The roles of factors regulating peroxisome fission (Figure 2) have not yet been systematically studied in flies. The primary effectors of peroxisome fission are Drp1, Fis1, Mff, and PEX11A/B (Schrader et al., 2016). *Drosophila* has homologs of all of these proteins (Figure 2). Increased levels of the Pex11 A/B homolog in S2 cells (Pex11) caused a two-fold increase in number and a corresponding reduction in volume (Baron et al., 2016). There are two predicted PEX11C homologs in *Drosophila*, (CG13827 and CG33474, Table 1). Other homologs of the peroxisome fission apparatus were identified independently as part of forward genetic screens for factors affecting mitochondrial proliferation. RNAi screens in S2 cells for regulators of mitochondrial fission identified *Tango11* as the homolog of mitochondrial fission factor (MFF, Figure 2) (Gandre-Babbe and van der Bliek, 2008). In human cells, the knockdown of MFF resulted in changes in peroxisome morphology similar to those observed for knockdown of dynamin-related protein (DRP, encoded by *DNM1L*) and hindered interaction of mitochondrial fission 1 protein (FIS1) with PEX11C (Figure 2) (Gandre-Babbe and van der Bliek, 2008; Koch and Brocard, 2012). Characterization of *Drp1* came specifically from employing flies to model human genetic disorders. A study using *Drosophila* was conducted to model the cellular impact of patients with infantile encephalopathy caused by *DNM1L* mutation rescued larval lethal *Drp1* mutants by expressing the human *DNM1L* homolog via transgenic insertion (Chao et al., 2016). In transgenic larvae expressing a dominant-negative *DNM1L* variant, peroxisomes in salivary glands were enlarged in volume and reduced in number (Koch et al., 2003; Chao et al., 2016; Assia Batzir et al., 2019). Overexpression of *Drosophila* Fis1 causes an increase in peroxisome number (Baron et al., 2016) but has not been otherwise characterized in terms of a role in peroxisome proliferation. However, Fis1 has been shown to play an analogous role in mitochondrial proliferation (Liu et al., 2012). The relationship between Pex11C and Fis1 also appears to be somewhat different in flies. In yeast, Pex11C is a positive regulator of peroxisomal fission (Chang et al., 2015), whereas overexpression of *Drosophila* PEX11C homolog CG13827 led to enlarged peroxisomes (Baron et al., 2016). Thus, the potential roles of Pex11 homologs in flies in terms of peroxisome fission (Figure 2) needs further exploration.

### Pexophagy

In each cell the biogenesis of peroxisomes is balanced by removal of old/excess peroxisomes. In mammalian cells, the population of peroxisomes is turned over approximately every 2 days (Huybrechts et al., 2009). In yeast or mammalian cells, defective or excess peroxisomes are removed by autophagy (pexophagy, reviewed in Germain and Kim, 2020). Some aspects of this process look to be conserved in *Drosophila*. Recently, HSPA9 (GRP75, Heat Shock Protein Family A Hsp70 Member 9) was identified as a suppressor of pexophagy in mammalian cells (Jo et al., 2020). The role is conserved in *Drosophila* as Gal4-UAS mediated RNAi of *Hsc70-*5, encoding the fly HSPA9 homolog also induced a reduction in the number of peroxisomes in third instar myocytes (Jo et al., 2020). Other aspects of pexophagy, including the effect of loss of the *autophagy related* (*ATG*) genes and their effect on fly peroxisomes has yet to be shown. Similarly, the role of *Drosophila* Pex proteins such as Pex2, Pex3, Pex11 or Pex14 or ubiquitination which have been shown to have roles in targeting peroxisomes for degradation in yeast or mammalian cells has not yet been examined directly.

### Other Pathways Regulating Peroxisome Volume or Number

Finally, the traditional fly forward genetic screen techniques are also uncovering other potential peroxisome regulatory factors in addition to those homologous to known regulatory factors in other species. A recent small-scale forward genetic screen in whole animals using number, volume and morphology of peroxisomes (marked by GFP-PTS1) as a readout (Graves et al., 2020) looked at only at the X-chromosome (∼15% of the fly genome). This screen identified a number of genes not previously implicated in peroxisome proliferation. Several genes encoding transcription factors not previously linked to peroxisomes such as: *fs(1)h*, *CG17829*, *mxc* or *Smox* were identified (Graves et al., 2020). These potential candidates may be acting analogously to PPARs or more likely represent independent pathways of peroxisome regulation. Of particular note, this screen only tested a limited number of genes on one chromosome and did not identify several known peroxisome regulators on the X such as *Pex5*, suggesting there are likely many additional novel peroxisome-linked genes to be identified using these methods.

While there are clearly some key differences in terms of regulation of proliferation and assembly factors, i.e., lack of a conserved PTS2 import pathway and no direct PPAR homologs, what is equally clear is that most peroxisome-linked genes and function are sufficiently conserved between *Drosophila* and mammals. There is similar conservation in mitochondrial regulatory pathways (reviewed in Sen and Cox, 2017) as well as in lipid metabolism (reviewed in Kühnlein, 2012). While these similarities support direct modeling of human peroxisome diseases in flies, the unexplored differences in flies will also likely provide valuable insight into novel aspects of peroxisome biogenesis and function.

## Conservation of Peroxisome Metabolic Activity in *Drosophila* Cells

The effects of mutation of *Pex* and other peroxisome linked genes on systemic metabolism and development provides some indication that peroxisome metabolism in *Drosophila* is more similar to that in mammals than to yeast. *Pex3* mutants had no detectable peroxisomes in their Malpighian tubule cells and died as larvae (Nakayama et al., 2011). *Pex3* mutants were hypersensitive to starvation with a strong reduction in lipid catabolism (Faust et al., 2014). Mutations in *Pex19* similarly showed reduced viability and dietary lipid sensitivity (Bulow et al., 2018). *Pex16* mutant adults have reduced body weight (Nakayama et al., 2011). *Pex2* and *Pex16* mutants were viable, but had increased sensitivity to glucose starvation suggesting reduced ability to metabolize lipids for energy (Wangler et al., 2017a). The number of peroxisomes in the Malpighian tubules is reduced in *Pex16* mutants and there is a two-fold elevation in VLCFA (>C24) levels compared to wild type. Mutations of *Pex16* does not affect circulating levels of MCFAs (<C18). There are viable and lethal mutations in *Pex10*. Viable *Pex10* males are sterile with defects in testes development. Adding VLCFAs to the diet, enhances the testes defect phenotype while addition of LCFAs has no effect (Chen et al., 2010). These same VLCFA-dependent testes phenotypes are also seen with *Pex14* mutations (Chen et al., 2010). *Pex5* mutants have an overabundance of VLCFAs compared to wild type flies (Di Cara et al., 2018, 2019). The somewhat specific response of Pex mutants in terms of VLCFA levels, supports a model that peroxisomes in flies are responsible for VLCFA metabolism, analogous to their role in mammalian cells.

### Management of Reactive Oxygen Species

A major peroxisome function is management of ROS. The linkage of *Drosophila* peroxisomal ROS-managing enzymes has largely been studied in the course of studying the effects of free radicals on aging. The activities of the antioxidant enzymes SOD, catalase, and glutathione reductase were examined across the lifespan of adult male *Drosophila*, and found to exhibit individual patterns of change with aging (Sohal et al., 1990). Measures of oxidative stress increased with age, as did total SOD activity, while catalase activity decreased sharply up to 10 days before death (Sohal et al., 1990). Over-expressing catalase and SOD in flies decreased overall oxidative stress, improving old-age fly activity and oxygen consumption rates, and enhancing longevity (Orr and Sohal, 1994). Similarly, the reduced longevity and motor deficits induced by peroxides generated from overexpression of the *Drosophila* homolog of *CG5009* (*ACOX1*) in wrapping glia was rescued by over-expression of catalase (Chung et al., 2020). Over-expressing human SOD1 in *Drosophila* motor neurons had a similar effect, suggesting age-related increases in oxidative stress had significant impact on nervous system function, and demonstrating conservation of SOD (Parkes et al., 1998a). Caloric restriction in flies was found to reduce peroxisome proliferation and improve longevity whereas mitochondrial ROS gene expression had the opposite effect (Zhou et al., 2012). Finally, Di Cara et al. (2018) did show direct effects on ROS management in S2 cells where *Pex5* has been targeted by RNAi. Together, these data suggested that the ROS management activities of peroxisomes were conserved between flies and humans. Further, it appears that *Drosophila* peroxisomes are the primary modulators of a diet-responsive oxidative state as tissue- and age-specific sensitivities to ROS were impacted in opposite ways in mitochondria and peroxisomes. From these initial studies, it may also be inferred that lipid metabolism, a major source of diet-related peroxisomal ROS in other species, is largely conserved between *Drosophila* and mammals.

### Lipid Oxidation

An analysis of the *Drosophila* proteome predicted multiple homologs for each of the five enzymes of the mammalian β-oxidation pathway, though none of the fly versions have been well characterized (Faust et al., 2012). Reporter chimeras of a predicted ACOX ortholg, CG17544 and Mtpα, co-localized with peroxisome marker PMP34-Cerulean in S2 cells. Several other predicted proteins had a variant PTS1 at their C-terminus, suggesting they also localized to peroxisomes (Faust et al., 2012). One predicted L-bifunctional protein, CG3415, had its quaternary structure resolved by X-ray scattering and was found to be very similar to that of human MFE-2, a known peroxisomal β-oxidation enzyme (Mehtala et al., 2013). A comprehensive screen of all predicted *Drosophila* peroxisomal proteins by Baron et al. (2016) found the most putative β-oxidation enzyme homologs identified by Faust et al. (2012) were greater than 75% co-localized with the peroxisome marker GFP-PTS1.

The functional roles for peroxisomes in terms of lipid β-oxidation in *Drosophila* appears to be similar to that of mammals, VLCFA levels were elevated in flies with *Pex2* or *Pex10* loss of function mutations (Chen et al., 2010). A metabolomics approach was used by Wangler et al. (2017a) to examine biochemical pathways affected by *Pex2* and *Pex16* mutation. They observed *Pex16* mutants were extremely sensitive to low sugar food, directly linking *Pex16* to glucose metabolism. Gas chromatography and mass spectrometry of *Pex16* mutants revealed changes in the abundance of C24, C26, C28, and C30 species of VLCFAs (Wangler et al., 2017a). Similarly *Pex5* mutant embryos showed an increase in VLCFAs, especially C24 (Di Cara et al., 2019).

A prerequisite for fatty acid β-oxidation is activation of fatty acids through ligation to coenzyme A (CoA), which is catalyzed by acyl-CoA synthetase (ACS; Krisans et al., 1980; Singh et al., 1992). *Drosophila* ACS, is encoded by the *Acsl* gene. Acsl was confirmed to regulate the conversion of VLCFA C16:1 to C16:1-CoA (Huang et al., 2016). *Acsl* loss resulted in overgrowth of neuromuscular junctions and increased phosphoethanolamine ceramide levels, the *Drosophila* equivalent of sphingomyelin, in motor neurons. These phenotypes were rescued by human *ACSL4* expression, establishing functional homology (Huang et al., 2016). There are several ACOX homologs in *Drosophila* although the closest homolog to ACOX1 is *CG5009*. Loss of activity of *CG5009* resulted in glial/axonal loss, reduced lifespan, impaired synaptic transmission and pupal death (Chung et al., 2020). These phenotypes were rescued by expression of human *ACOX1*. This strongly suggested *Drosophila acyl-Coenzyme A oxidase at 57D* genes are conserved in terms of the rate-limiting, peroxide-producing, peroxisomal-localized first step of VLCFA β-oxidation performed by their mammalian counterpart. The remaining homologs of peroxisomal lipid oxidation enzymes identified by Faust et al. (2012) await functional characterization in terms of their role in lipid metabolism.

Bulow et al. (2018) showed that homozygous *Pex19* mutation results in an increase in free fatty acids, leading to lipotoxicity via altered abundance in MCFAs due to down-regulated mitochondrial lipolysis. Restoring MCFA abundance by dietary supplementation rescued the phenotype in flies and *PEX19* patient cells (Sellin et al., 2018). Similar changes in MCFA abundance and altered mitochondrial lipolysis were observed in *Pex2*, *Pex3* and *Drp1* mutant larvae (Chao et al., 2016; Bulow et al., 2018; Sellin et al., 2018). In the case of *Drp1* mutants, aberrant mitochondrial morphology was observed in nervous and muscle tissue (Chao et al., 2016). Given the changes in lipid metabolism observed in *Pex* mutants, it would seem that the roles for peroxisomes and mitochondria in terms of β-oxidation in *Drosophila* appear to be more similar to mammals than to yeast.

### Lipid Synthesis

What is known regarding the conservation of this peroxisome activity in *Drosophila* lipid synthesis activity is limited largely to sequence-based homology prediction of the constituent enzymes. The exception is the cytosolic DHAP reductase/GPD1 homolog Gpdh1, whose oxidase (dehydrogenase) activity has been characterized (O’Brien and Macintyre, 1972; Niesel et al., 1980). The ability of Gpdh1 to reduce DHAP, as required by the ether lipid synthesis pathway, has not been explicitly observed in *Drosophila*. The human homolog GPD1 does have this capability (Reyes et al., 2015). Several homologs were predicted for other mammalian ether lipid synthesis enzymes by Faust et al. (2012). One of these was the screen for factors that affect *Drosophila* response to viral infection identified the potential *FAR* homolog *Sgroppino* described above (Faust et al., 2012; Merkling et al., 2019). FAR proteins function by reducing fatty acyl-CoA into a fatty alcohol molecule, however, this function has yet to be directly confirmed in *Drosophila*. What has been shown is that loss of *Sgroppino* interfered with β-oxidation in adults resulting in increased levels of triacylglycerol (TAG) and increased body mass (Merkling et al., 2019). Similarly, trafficking of Gpdh1 to peroxisomes has not been confirmed, though a variant PTS1 was identified at the C-terminus of Gpdh1 (Wojtas et al., 1997).

## Developmental Defects Linked to Peroxisome Dysfunction in *Drosophila*

There seems to be a varied phenotypic spectrum associated with mutations in *Pex* and other peroxisome-linked genes during development. The mRNA encoding most *Pex* genes is expressed ubiquitously during early embryo development, although some (*Pex7*, *Pex13*, *Pex14*, *Pex19*) display differences in relative mRNA levels between various cell lineages (Leader et al., 2018; Pridie and Simmonds, 2020). However, likely due to a large maternal contribution of peroxisomes, fly embryos develop into larvae even when homozygous for most *Pex* gene mutations. Embryos homozygous for a *Pex1* null mutation had a severe effect on larval growth when the animals were raised on a diet with yeast as the primary food source (Mast et al., 2011). *Pex1* mutants also have defects in sperm production (Chen et al., 2010). However, insertion of a transposable element into *Pex6* does not affect viability or fertility (Bellen et al., 2004). Loss of *Pex3* eliminates peroxisome formation and mutants die before pupariation (Nakayama et al., 2011). An insertional mutation of *Pex5* is lethal at or before pupariation with increased cell death. *Pex7* mutants are viable (Di Cara et al., 2019; Pridie and Simmonds, 2020). Mutations of genes encoding proteins of the importomer *Pex2*, *Pex10* and *Pex12* are viable but are associated with sterility phenotypes. Flies with *Pex13* mutations survive to adult although there are some effects on fertility (Chen et al., 2010). Currently no mutations of *Pex14* are available. *PEX16* is essential for peroxisome biogenesis in humans, *Pex16* maternal zygotic mutant embryos retained some peroxisome-like structures into the larval stage while over-expression of *Pex16* led to fewer and larger peroxisomes (Nakayama et al., 2011). The major developmental phenotypes of *Pex16* loss are male sterility and the *ry* eye phenotype (Nakayama et al., 2011). Similar to *Pex5*, *Pex19* mutant flies die before the end of the larval stages, with some reaching adulthood. However, if *Pex19* activity is eliminated in the oocyte and the zygote then mutants die as embryos (Bulow et al., 2018).

### Mutations Affecting Potential Peroxisome Fission Factors

Mutations affecting potential peroxisome fission factors (Figure 2) have not been well characterized. Mutation or dsRNA knockdown of *Pex11*, *CG13827* or *CG33474* are largely viable and fertile (Thurmond et al., 2019). The only reported phenotype is mild defects in sensory bristle patterning with tissue-specific Gal4/UAS driven expression of dsRNAs targeting *CG13827* (Mummery-Widmer et al., 2009). Mutations in *Drp1* and Tango11 cause lethality before the end of larval stages and targeted RNAi in neurons leads to similar alterations in bristle morphology as CG13827 altered mitochondrial morphology (Mummery-Widmer et al., 2009; Zhou et al., 2019). Targeted expression of dsRNAs targeting Fis1 in motor neurons leads to defects in axonal transport (Liu et al., 2012).

### Peroxisome Membrane Proteins

There are relatively few mutants of genes encoding fly homologs of peroxisome membrane proteins (Table 2) and most UAS-RNAi lines or existing mutants are viable (Thurmond et al., 2019). There are several mutants for *ABCD* which are viable and fertile, but targeted expression of UAS-RNAi targeting *ABCD* causes defects in the developing CNS and retina (Gordon et al., 2018). Expression of UAS-RNAi constructs targeting *Orct* in the fat body in third instar larvae cause a lipid storage phenotype (Fan et al., 2017). Similarly, there are few available mutants in genes encoding homologs of peroxisome enzymes (Tables 3, 4). Of those that are available, most are viable and fertile (Thurmond et al., 2019). However, mutations in *Cat, Hmgcr, Acsl*, *Lon* or *Sod1* have been isolated and all are lethal although in the case of *Lon*, this may be caused by effects on mitochondrial function (Griswold et al., 1993; Perrimon et al., 1996; Parkes et al., 1998b; Zhang et al., 2009; Liu et al., 2014; Pareek et al., 2018). Flies with homozygous recessive mutations in *Ascl* or *Nos* have CNS defects (Zhang et al., 2009; Liu et al., 2014; Rabinovich et al., 2016) and *Mfe2* mutants cause a small larva phenotype (Zhou et al., 2019). Finally, flies with homozygous *Cat*, *Mul1* and *Mtp*α mutations are viable but have a reduced lifespan (Missirlis et al., 2003; Kishita et al., 2012; Doktór et al., 2019). Generally developmental defects associated with *Pex* gene mutations in *Drosophila* appear to be most prevalent in the CNS and PNS, gonad, innate immunity, gut development/repair as well as an interesting link to aging.

### Neural/Neuromuscular Development

Neural development is strongly affected in flies with *Pex* gene mutations. Several neuronal lineages showed disorganized structure in the developing *Pex1* mutant embryo (Mast et al., 2011). *Pex5* mutants showed defects in the embryonic CNS (Di Cara et al., 2019). Although loss of *Pex19* function is generally lethal, rare adults do survive (escapers). These adult eascapers had elevated apoptotic activity in their optic lobes, suggesting neurodegeneration, as well as poor negative geotaxis and an inability to fly or inflate their wings (Bulow et al., 2018). *Pex3* RNAi and loss-of-function mutation resulted in a lethal partially eclosed (dead) phenotype. This inability to break out of the pupal case was traced to defects in adult muscle function, thought to be due to defect neuromuscular junction activity (Faust et al., 2014). Notably, loss of *Acsl* resulted in the same phenotypes as *Pex3* mutation, however, the mechanism was traced to compromised neuromuscular junctions due to neurodegeneration of motor neurons and glia, rather than musculature defects (Huang et al., 2016).

Neuronal degeneration is also seen with mutations affecting ACS function in flies. There are two *Drosophila* genes encoding homologs of the human ACS protein family, *bubblegum* (*bgm*) and *heimdall* (*hll*, aka *doublebubble*). Both are most similar to human ACS bubblegum family member 2 protein. *bgm*/*hll* double knockout resulted in animals with neurodegeneration and VLCFA accumulation somewhat similar to that of adrenoleukodystrophy (Wiesinger et al., 2013; Sivachenko et al., 2016). Activated fatty acid molecules, such as very long-chain acyl-CoA ester, are a substrate for the fatty acid transporter ATP-binding cassette sub-family D member 1 (ABCD1; Wiesinger et al., 2013). Knockdown of the potential *Drosophila* ABCD1/2 homolog showed optic lobe neurodegeneration very similar to that observed in the *Bgm*/*Hll* double knockout strain (reviewed in Gordon et al., 2018).

Given that mutation in many *Pex* and peroxisome enzyme genes cause CNS/PNS defects, an intriguing possibility is that ROS is used commonly as a signaling molecule during development. The role of ROS as a paracrine signal in CNS/PNS development has not been shown. However, ROS is used as a paracrine signal in at least one fly cell lineage, the cardiomyocyte (Lim et al., 2014). The adult *Drosophila* heart is a linear tube made of two columns of cardiomyocytes (CMs) encased in a sheath of non-muscle pericardial cells (PCs) called a pericardium. In mammals there is cross-talk between the various PC and CM subtypes that is essential for the heart to respond to physiological and pathological cues (reviewed in Tian and Morrisey, 2012). Elements of that relationship are preserved in *Drosophila*, as PCs influence both myocardial development and heart function (Lim et al., 2014). Adult PCs have higher ROS abundance than CMs *in vivo*. ROS reduction in PCs resulted in increased arrhythmia (Lim et al., 2014). Reducing ROS activity by increased expression of Catalase or Sod1 lead to hearts that were narrower at 1 week post-eclosion, and by 4 weeks post-eclosion the diastolic diameter had become so great that the heart tubes were enlarged (Lim et al., 2014). The phenotype was not observed when ROS levels were altered in CMs, nor did altering PC ROS levels affect CM ROS levels, indicating the phenotype was not due to ROS diffusion from PC-to-CM (Lim et al., 2014). A possibility that will need to be considered is the use of ROS as a paracrine signal in fly development. The ROS-mediated paracrine signaling observed in cardiomyocytes, which would likely involve peroxisome activity and it will be particularly interesting to see if similar mechanisms are involved in cell signaling in the developing CNS/PNS or other organs.

### Gonad Development

Homozygous *Pex2*, *Pex10* and *Pex12* null mutants were viable and survived to adulthood, however, males were sterile and females had reduced fertility. This was caused by a defect in sperm development correlated to increased VLCFA abundance, which was proposed to cause a failure in cytokinesis during meiosis (Chen et al., 2010). Adult *Pex16* mutant males also had the same sterility phenotype as *Pex2* and *Pex10* adult males, though the phenotypic defect was traced to arrested spermatocyte development, not cytokinesis failure (Nakayama et al., 2011). Given the sterility phenotypes associated with these *Pex* genes, it would seem that multiple steps of sperm development are acutely sensitive to fatty acid levels regulated by peroxisomes. At this point, little is known about the specific mechanistic requirements for peroxisome activity in the *Drosophila* ovaries. However, it has been shown recently that there are distinct differences in TAG metabolism between male and female flies (Wat et al., 2020). Whether there are corresponding sex-specific differences in peroxisome activity, especially in the gonads, remains to be determined.

### Immunity

In the *Drosophila* circulatory system, hemocytes circulate in a blood-like fluid called hemolymph. Circulating hemocytes act analogously to mammalian macrophages as the principal effectors of the *Drosophila* immune response (reviewed in Parsons and Foley, 2016). In mammals, when a macrophage initiates phagocytosis it releases ROS to damage the pathogen being engulfed (reviewed in Thomas, 2017). *Drosophila* S2 cells are thought to be of hemocyte lineage (Cherbas et al., 2011). Peroxisomes in S2 cells were observed associating with phagosomes surrounding *E. coli* actively moving to the site of the nascent phagocytic cup (Di Cara et al., 2017). RNAi knockdown of *Pex5* or *Pex7* compromised uptake of *E. coli* and *C. albicans* due to defects in actin organization surrounding the phagocytic cup, and defects in ROS management. Over-expression of *Catalase* rescued phagocytosis in cells treated with dsRNA targeting *Pex7*, but not when *Pex5* was targeted (Di Cara et al., 2017). This same effect was observed in primary hemocytes. Adult flies with hemocyte-specific Gal4/UAS expression dsRNAs targeting *Pex5* or *Pex7* mRNAs died within 7 days of infection. The *Pex5* RNAi-treated flies were also more sensitive to injury (Di Cara et al., 2017). A similar response was seen adult *Drosophila* midgut microbial response when *Pex5* was RNAi targeted resulted (Di Cara et al., 2018).

### Gut Development and Repair

Studies in *Drosophila* have shown clearly that peroxisome function is essential to development and tissue homeostasis in the intestine. Gal4/UAS mediated expression of *Pex5* dsRNAs in these organs resulted in lethally high levels of autophagy due to AMPK-dependent TOR kinase inhibition (Di Cara et al., 2018). Gut epithelia with dysfunctional peroxisomes had increased abundance of non-esterified fatty acids and increased redox stress, severely disrupting gut homeostasis (Di Cara et al., 2018). Loss of *Pex2* or *Pex10* in the *Drosophila* gut blocked intestinal stem (progenitor) cells from maturing into mature enterocytes in damaged midgut (Du et al., 2020). Through a combination of mutant analysis, Gal4/UAS mediated transgene expression and dsRNA knockdown Du et al. (2020) showed that peroxisomes are required in a cell autonomous manner for stem cell differentiation. Comparitive mRNA-seq identified the likely mechanism for increased peroxisome numbers in the midgut was an enhancement in RAB-7 maturation to promote stem cell/progenitor cell differentiation through the JAK/STAT-SOX21A signaling pathway (Du et al., 2020). Notably, this requirement for increased peroxisome activity for repair of damaged intestinal epithelia in *Drosophila* is conserved in mice and humans as well (Du et al., 2020). This suggests a potentiality that peroxisomes, along with mitochondria and other organelles like the endosomal pathway coordinately integrate lipid metabolism with other internal and external metabolic signals in the gut to maintain organ homeostasis, especially after injury (reviewed in Bellec and Cordero, 2020).

### Aging

Ectopic *Catalase* over-expression in mitochondria and the cytosol were found to increase adult *Drosophila* oxidative stress resistance but not improve longevity (Mockett et al., 2003). In terms of system-wide changes, flies have proven to be particularly amenable to RNA expression RNA-Seq of dietary restricted flies with enhanced lifespan compared to wild type flies showed elevated expression of *Catalase* (*Cat*), *Pex16*, *ry*, *Dodecenoyl-CoA delta-isomerase* (*Dci*) and *Sod* (Li et al., 2019). A similar experiment showed changes in the relative levels of *Pex1*, *Pex2*, *Pex5*, *Pex6*, *Pex7*, *Pex10*, *Pex11*, *Pex16*, and *Pex19* mRNA in aged flies compared to those treated with Paraquat, a herbicide that strongly induces ROS (Huang et al., 2019). Together, these findings suggest enhanced peroxisome biogenesis and up-regulated expression of peroxisomal ROS management enzymes improved longevity, whereas increased mitochondrial ROS gene expression only improved tolerance to oxidative stress, i.e., the ROS generated by oxidative phosphorylation. Like what was shown in terms of gut homeostasis, peroxisome function plays a role in aging-related inflammation induced cardiac disease. Huang et al. (2020) showed that reduction in peroxisome protein import in oenocytes, either experimentally by reduction of factors regulating peroxisome import of Pex1, Pex5, or Pex14 activity, disrupted ROS homeostasis cause by Paraquat treatment or due to aging, led to cardiac arrhythmia (Huang et al., 2020). Notably, the age-related effect on the heart could be suppressed by Gal4 > UAS-Pex5 expression in the oenocytes and that the effect was mediated by induction of the cytokine Unpaired 3, a JAK/STAT ligand (Hombría et al., 2005). Again, this newly discovered role for peroxisome function seems to be conserved between flies and mammals (Huang et al., 2020).

There are likely multiple yet-to-be discovered roles for peroxisomes in integrating multiple signals to help mediate tissue homeostasis. The mechanisms identified so far range from regulating ROS or lipid metabolism to more direct effects on secretion of cell-signaling molecules. Notably, sex-specific differences peroxisome-linked effects on aging have been identified. Female flies with non-lethal mutations in *Pex1* or *Pex13* had reduced peroxisome proliferation, reduced cellular peroxide levels and, paradoxically, increased lifespan compared to males (Zhou et al., 2012). What will be particularly interesting as this field develops is understanding the coordination between peroxisomes and other organelles like mitochondria, lipid droplets, the endosomal pathway and lysosomes in mediating these coordinating events in the specialized cells that make up these different organs. Knockdown of Pex13 in the fat body by RNAi causes mitochondrial fragmentation supporting functional linkage between mitochondria and peroxisomes (Zhou et al., 2019). Similarly, it will be interesting to see if peroxisomes in these cells have evolved correspondingly specialized activities, including sex-specific differences.

## Measuring the Dynamic Movement of Peroxisome Movement in S2 Cells

The study of peroxisome movement in S2 cells, is distinct from the functional characterization of peroxisomes as described above. S2 cells have been used to study *Drosophila* protein localization to peroxisomes as well as peroxisome metabolism or function in microbial phagocytosis. However, unique aspects of their microtubule organization has been leveraged to study peroxisome transport on the cytoskeleton. In yeast and mammalian cells, peroxisomes are subject to constant relocation involving dynamic elements of the cytoskeleton (Subramani, 1998). Electron microscopy of Chinese hamster ovary cells revealed multiple peroxisome-microtubule contact sites (Rapp et al., 1996). *In vivo* kinetic analysis determined chemical disruption of both microtubule and actin filaments reduced peroxisome movement, as did ATP depletion, implying peroxisomes were moved by motor proteins (Rapp et al., 1996). *Drosophila* peroxisomes appear to move along microtubules like they do in mammalian cells, rather than the actin/myosin transport mechanism characteristic of yeast peroxisomes (reviewed in Neuhaus et al., 2016).

The establishment of a S2 cell line stably expressing GFP-PTS1, has become a model of choice for *in vivo* peroxisome dynamics studies. When S2 cells are grown on surfaces coated and treated with Cytochalasin D they extend processes less than one micrometer in diameter with bundles of microtubules orientated in one direction. Combined, these two approaches allow extremely precise measurement of the movement of peroxisomes along microtubules in live cells (Kural et al., 2005). Using this system Kinesin-1 and Dynein heavy chain were found to be the motor proteins responsible for salutatory peroxisome movement along microtubules in *Drosophila* (Kural et al., 2005). Peroxisome-associated microtubules contributed to individual organelle movement in a motor protein-dependent manner, and opposite-polarity motors interacted to promote bi-directional peroxisome movement (Kulic et al., 2008; Ally et al., 2009). Kinesin heavy chain anchors Kinesin-1 to peroxisomes, and thus regulates retrograde peroxisome movement. Using single particle tracking of peroxisomes at millisecond time resolution, the saltatory movement of peroxisomes via a plus-minus motor tug of war along microtubules was observed. The precise measurements facilitated by the microtubule organization of S2 cells showed that the saltatory pattern was due to cross-linking to multiple microtubules which communicated via the peroxisome membrane (De Rossi et al., 2017). S2 cell studies are also starting shed light on other potential regulatory factors affecting peroxisome movement on microtubules. A whole-genome dsRNA screen for factors affecting peroxisome transport found that the Ser/Thr kinase Darkener of Apricot, bound to eukaryotic translation elongation factor 1γ, were critical regulators of microtubule-based transport of peroxisomes (Serpinskaya et al., 2014).

## Summary: A Rosy Future for Flies as a Model System to Study Peroxisomal Disorders

The localization and function of most *Drosophila* peroxisome protein homologs has been validated experimentally, in S2 cells and in many cases in whole animals. However, there are still several enzymes where a peroxisomal localization has not been validated experimentally, especially those that do not have a canonical PTS1 targeting sequence (Table 4). It may be that these enzymes are cytoplasmic and metabolites pass in and out of the peroxisome or alternatively, it may be that the tagging approach used by Baron et al. (2016) interfered with localization. Validation of a peroxisomal role/localization will require alternative approaches. Similarly, there a few missing homologs to key enzymatic pathway steps known to occur in peroxisomes in yeast in humans. It may be that other proteins can substitute functionally or alternatively that these steps are missing in flies. Screens for metabolic requirements, focused on peroxisomal function would help clarify these missing steps.

*Drosophila* screens have identified several novel aspects of peroxisome genes that need further examination. One example of this is a potential role for localized regulation of translation of mRNAs encoding peroxisome proteins. Fly embryos are strongly dependent on regulated mRNA localization and translation. The first 90 min of embryo development occurs largely without zygotic gene transcription but requires a large store of energy stored as lipid in the yolk, which would likely maintenance of the peroxisome population via proliferation and pexophagy. Many *Pex* mRNAs show highly specific, often punctate, patterns in the large cytoplasm of the oocyte or syncytial early embryo suggesting post-transcriptional gene regulation (reviewed in Hughes and Simmonds, 2019).

Another major unanswered question is the variable requirement/activities of peroxisomes in different organs/cell lineages. Notably, patients with Pex mutations often have variable defects in organ development or function, especially the CNS (reviewed in Faust et al., 2005). The simple body plan and CNS/PNS structure of fly larvae and adults, combined with the facile Gal4/UAS mediated tissue-specific gene targeting provides a facile platform to begin to dissect the requirements for peroxisome activity in general in the CNS and other organs. The same approach can be used to probe the tissue-specific effects of mutations in *Pex* genes or those encoding specific peroxisome enzymes. Flies also represent an excellent animal to examine the coordinated roles of peroxisomes, mitochondria and lipid droplets in terms of lipid homeostasis. Notably, mutation or tissue-specific knockdown of gene encoding enzymes like Mtpα, Orct or CG9527 (ACOX) that localize to fly peroxisomes lead to changes in the size or morphology of lipid droplets. Similarly, targeted knockdown or mutation of *Pex1*, *Pex13*, *Pex19*, *Crat, Mfe2* and *Mul1* lead to changes in mitochondrial morphology. The linkage between peroxisome activity and mitochondrial function is particularly intriguing given the discovery that MCFAs can suppress the effects of Pex19 mutation, including those effect on mitochondria (Sellin et al., 2018).

Helping to answer these and other novel aspects of fly peroxisomes will be new reagents/technologies becoming available to study peroxisomes in flies. Community-wide efforts have generated extensive collections of new *Pex* and other peroxisome gene mutants which are then made available to the greater academic research community. A good example is the TRiP Toolbox generated by the Harvard Medical School DRSC/TRiP Functional Genomics Resources group (Perkins et al., 2015; Hu et al., 2017; Zirin et al., 2020). The TRiP Toolbox combines the targeting capacity of the Gal4-UAS binary expression system with lines expressing specific gene targeting dsRNAs as well as CRISPR-Cas9 to produce targeted *in vivo* mutagenesis of a gene of interest (reviewed in Lin et al., 2015). This system can be used for targeted gene knockout (TRiP-KO) or, using a deactivated variant of Cas9 (dCas9), targeted gene over-expression (TRiP-OE). Similarly, the collection of tissue-targeted dsRNA expressing UAS-lines continues to expand, providing a simple and effective way to knock down peroxisome-linked genes in specific tissues or to express modified forms of these genes mirroring human mutations. Similarly, it is relatively easy to profile fatty acids in a tissue or whole organism basis, further supporting the ease of use of *Drosophila* for studying metabolic disorders (Parisi et al., 2011; Carvalho et al., 2012; Sellin et al., 2020). This utility of flies to explore the pathophysiological effects of genes linked to rare genetic diseases, including the developmental defects associated with peroxisome disorders is now supported by multiple groups worldwide. Some examples of these consortiums include: the Undiagnosed Diseases Network, the Centers for Mendelian Genomics and the Canadian Rare Diseases Models and Mechanisms Network (Ramoni et al., 2017; Posey et al., 2019; Boycott et al., 2020; Taruscio et al., 2020); reviewed in Wangler et al. (2017b). The unique aspects of fly development, ample genetic tools and a large support system of technological advances and reagents will continue to provide new avenues of exploration to improve our understanding of peroxisomes. Thus, the ‘re’-emergence of flies as an effective and facile model system for studying peroxisomes portends a ‘rosy future.’

## Author Contributions

All authors contributed substantially in terms of the concept and content of this review.

## Conflict of Interest

The authors declare that the research was conducted in the absence of any commercial or financial relationships that could be construed as a potential conflict of interest.

## Footnotes

1 http://flybi.hms.harvard.edu

## Abbreviations

ALS: amyotrophic lateral sclerosis
CNS: central nervous system
LCFA: long-chain fatty acid
MCFA: medium chain fatty acid
PNS: peripheral nervous system
PTS: peroxisome targeting signal
ROS: reactive oxygen species
UAS: upstream activating sequence
VLCFA: very long-chain fatty acid.
